# Ultrasound-Guided PECS II + Serratus Plane Fascial Blocks Are Associated with Reduced Opioid Consumption and Lengths of Stay for Minimally Invasive Cardiac Surgery: An Observational Retrospective Study

**DOI:** 10.3390/life12060805

**Published:** 2022-05-28

**Authors:** Debora Emanuela Torre, Carmelo Pirri, Marialuisa Contristano, Astrid Ursula Behr, Raffaele De Caro, Carla Stecco

**Affiliations:** 1 Department of Cardiac Anesthesia and Intensive Care Unit, Cardiac Surgery, Ospedale dell’Angelo, 30174 Venice Mestre, Italy; t_debora3108@hotmail.it; 2 Department of Neurosciences, Institute of Human Anatomy, University of Padova, 35121 Padova, Italy; rdecaro@unipd.it (R.D.C.); carla.stecco@unipd.it (C.S.); 3 ICLAS, GVM, Istitituto Clinico Ligure, Alta Specialità, 16035 Rapallo, Italy; contristano@hotmail.it; 4 Operative Unit of Anesthesia and Resuscitation, Hospital of Camposampiero, 35012 Camposampiero, Italy; astridursula.behr@gmail.com

**Keywords:** regional anesthesia, minithoracotomy, cardiac surgery, pain, PECS II block, serratus plane block, opioids sparing, fascia, ERAS

## Abstract

This study tested the hypothesis that pectoralis II (PECS II) + serratus plane blocks would reduce opioid consumption and improve outcomes compared with standard practice in minimally invasive cardiac surgery. A retrospective and observational study was realized in the intensive care unit (ICU) setting of “ICLAS GVM, Istitituto Clinico Ligure Alta Specialità, (Rapallo, Italy)”, including adult patients who underwent right minithoracotomy for replacement/plastic aortic, mitral and tricuspid valve or atrial myxoma resection in cardiac surgery. Seventy-eight patients were extracted by the database and divided into two groups. Group 1 (41 patients) received ultrasound-guided PECS II + serratus plane blocks with Ropivacaine 0.25% 10 mL + 20 mL + 30 mL. Group 2 (37 patients) received intravenous opioids analgesia with morphine 20–25 mg/day or tramadol 200–300 mg/day. The primary outcomes were: the pain perceived: Critical-Care Pain Observation Tool (CPOT) score; the opioids consumption: mg morphine or tramadol, or µg sufentanyl administered; and mg paracetamol, toradol, tramadol or morphine administered as a rescue. The secondary outcomes were the hours of orotracheal intubation and of stay in ICU, and the number of episodes of nausea, vomiting, delayed awakening and respiratory depression. Group 1 vs. Group 2 consumed less opioids (Sufentanyl *p* < 0.0001; Morphine *p* < 0.0001), had a lower pain perceived (*p* = 0.002 at 6 h, *p* = 0.0088 at 12 h, *p* < 0.0001 at 24 h), need for rescue analgesia (*p* = 0.0005), episodes of nausea and vomiting (*p* = 0.0237) and intubation time and ICU stay (*p* = 0.0147 time of IOT, *p* < 0.0001 stay in ICU). Ultrasound-guided PECS II + serratus plane blocks demonstrated better than intravenous opioids analgesia in patients undergoing minimally invasive cardiac surgery.

## 1. Introduction

Minithoracotomy, in minimally invasive cardiac surgery, is associated with high postoperative pain [1,2]. For pain management in many surgeries, Enhanced Recovery After Surgery (ERAS) protocols recommend opioid-sparing, the use of multimodal analgesia and the use of local analgesia. For example, in liver surgery, they recommend the use of transversus abdominis plane (TAP) block or paravertebral block [3]; in orthopedic surgery (in total hip or knee arthroplasty), they recommend the femoral block or the Hunter canal block [4]; in colorectal surgery, they recommend peridural analgesia or TAP block [5]; in thoracic surgery, they recommend the serratus plane block or the peridural analgesia [6]. In ERAS protocols of cardiac surgery, in the chapter “pain management”, they also recommend opioid-sparing for their collateral effects (nausea, vomiting, ileus, delayed awakening, respiratory depression, prolonged time of stay in intensive care unit (ICU), prolonged time of intubation) and the use of multimodal analgesia (tramadol, acetaminophen and NSAIDs, dexmedetomidine, etc.) but they do not recommend regional anesthesia/analgesia for the pain management [7]. There are several regional analgesia options for cardiac surgery pain management: peridural analgesia, paravertebral block, erector spinae plane (ESP) block, serratus plane block and PECS II block. These techniques were enhanced by comprehensive utilization of ultrasound guidance, the latter being cost-effective, readily accessible, reliable imaging and having multiple advantages [8,9,10,11,12,13].

PECSII block proved part of a postoperative multimodal strategy in patients undergoing cardiac surgery with sternotomy [8]. Serratus plane block was studied in minimally invasive heart valve surgery with right thoracotomy and minimally invasive direct coronary artery bypass with left thoracotomy [9]. PECS II block and serratus plane block (SAP block) are more realizable together because the patient is lying down in a supine position in an intensive care unit (ICU) bed after cardiac surgery.

In September 2019, the postoperative pain management protocol for patients undergoing minithoracotomy in cardiac surgery changed from routine use of opioids alone to the use of opioids alone or PECS II + SAP blocks based on the skills of the anesthesiologists. By taking all data into account, the purpose of the study was to investigate whether the use of PECS II block + serratus plane block improved the pain perceived, the need for rescue analgesia, the Critical-Care Pain Observation Tool (CPOT) score, the opioids sparing, the intubation time, the ICU stay and collateral opioid-related effects (episodes of nausea and vomiting, delayed awakening, respiratory depression), compared to the intravenous opioids’ analgesia.

## 2. Materials and Methods

### 2.1. Study Design, Population and Data Sources

This is an observational, retrospective, single-center study including adult patients who underwent right minithoracotomy for replacement/plastic aortic, mitral and tricuspid valve or atrial myxoma resection in cardiac surgery between November 2019 and March 2020. All data were extracted from “ICLAS, GVM, Istitituto Clinico Ligure, alta specialità, (Rapallo, Genova, Italy)” database. The study was approved by the appropriate Local Ethics Committee, and informed consent was obtained from all subjects involved in the study. Data were collected anonymously using a standardized data collection form. The exclusion criteria were: patients less than 18 years of age, repeated cardiac surgery, opioid use and substance abuse. The authors categorized the patients into two groups and changed the protocol for the patients who underwent minithoracotomy in cardiac surgery, from routine use of opioids alone to the use of opioids alone or PECS II + SAP blocks, based on the skills of the anesthesiologist. Patients that received PECS2 block + serratus plane block with Ropivacaine 0.25% 10 mL + 20 mL + 30 mL were considered for inclusion in group 1, whereas group 2 included the patients that received intravenous opioids analgesia with morphine 20–25 mg/day (1.2 mg/h increased or decreased pain perceived) or tramadol 200–300 mg/day.

The data collected consisted of the patient’s sex; age; BMI; height; weight; starting ejection fraction; type of surgery and drainage position; CPOT score after 30 min, 1 h, 2 h, 6 h, 12 h, 24 h of the start of analgesia (locoregional or intravenous); high CPOT score rescue analgesia administration; total consumption of sufentanyl, morphine, tramadol, toradol and paracetamol; number episodes of nausea and vomiting; delayed awakening; respiratory depression; time of intubation; and time of stay in ICU.

### 2.2. Primary and Secondary Outcomes

The primary outcome was the pain perceived assessed using the CPOT score and the opioids consumption evaluating mg of morphine or tramadol or sufentanyl administered; the milligrams of paracetamol, toradol, tramadol or morphine administered as rescue were also assessed.

The secondary outcomes were the hours of orotracheal intubation and the hours of stay in ICU, the number of episodes of nausea and vomiting, delayed awakening and respiratory depression.

### 2.3. Pre- and Intra-Operative Management

**Figure 1:**
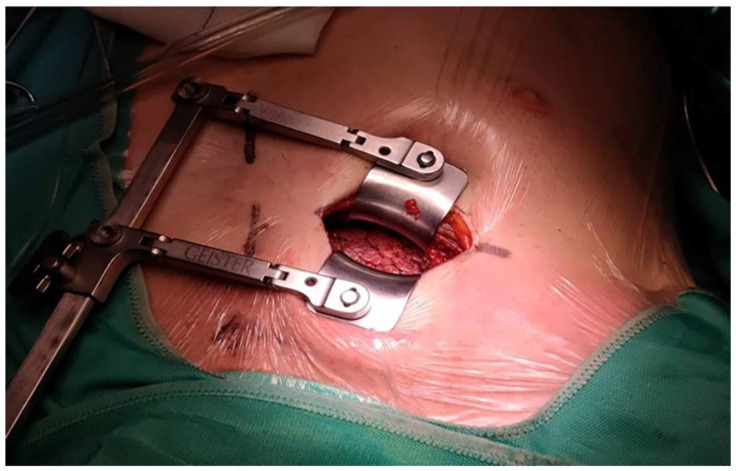
Right minithoracotomy, 6–8 cm surgery incision between 2nd and 3rd intercostal space.

Induction and maintaining anesthesia were the same into two groups: midazolam 0.15 mg/kg, sufentanyl 0.6 µg/kg and cisatracurim (0.2 mg/kg) or rocuronium (0.6 mg/kg) for induction of anesthesia; propofol 4 mg/kg/h and sufentanyl 0.5 µg/kg/h for maintenance. Right minithoracotomy was a 6–8 cm surgical incision between the 2nd and 3rd intercostal space (in aortic valve surgery) (Figure 1), between the 3rd and 4th intercostal space (in mitral and tricuspid valve surgery, and in atrial myxoma resection). In the end, the surgeon placed chest drainages in the 2nd and 3rd intercostal space on the median axillary line in aortic valve surgery, in the 3rd and 4th intercostal space on the median axillary line in mitral and tricuspid surgery and in atrial myxoma resection.

For group 1, at the patients’ admission to ICU, the blocks were performed.

#### 2.3.1. PECS II Block

**Figure 2:**
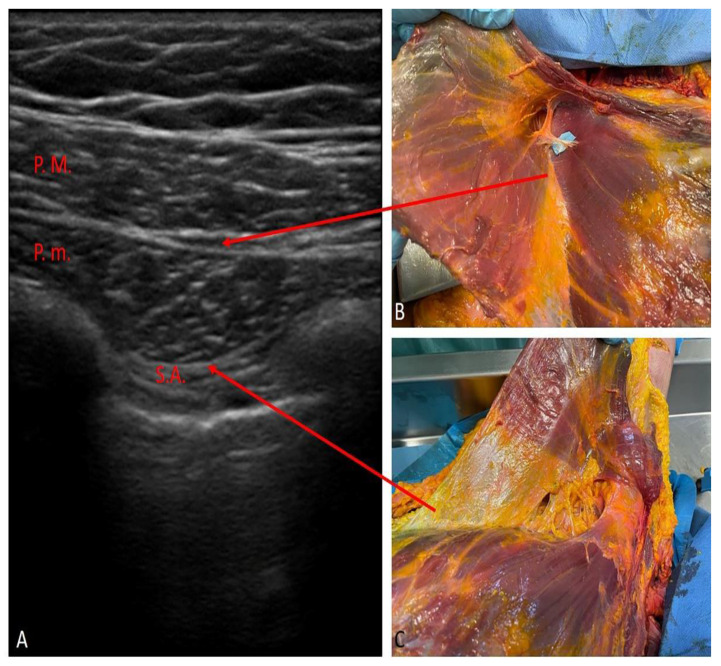
PECS II block: (**A**) oblique ultrasound imaging scan along the medioclavicular line 2nd rib level; P.M.—pectoralis major muscle; P.m.—pectoralis minor muscle; S.A.—serratus anterior muscle. (**B**,**C**) anatomical correlation dissections of the sites of inter-fascial injections.

The PECS II block was performed using a Stimuplex Braun 22 G × 80 mm echogenic needle, a linear ultrasound probe (4–12 MHz) and an ultrasound PHILIPS machine. An amount of 10 mL Ropivacaine 0.25% was injected in the inter-fascial plane between the pectoralis major and minor muscles at the 2nd cost level (Figure 2A,B), and 20 mL Ropivacaine 0.25% in the interfascial plane between pectoralis minor and serratus muscle at 3rd/4th costal level (Figure 2A–C). The patient was in a supine position with the arm near the chest or in 90° abduction.

#### 2.3.2. SAP Block

**Figure 3:**
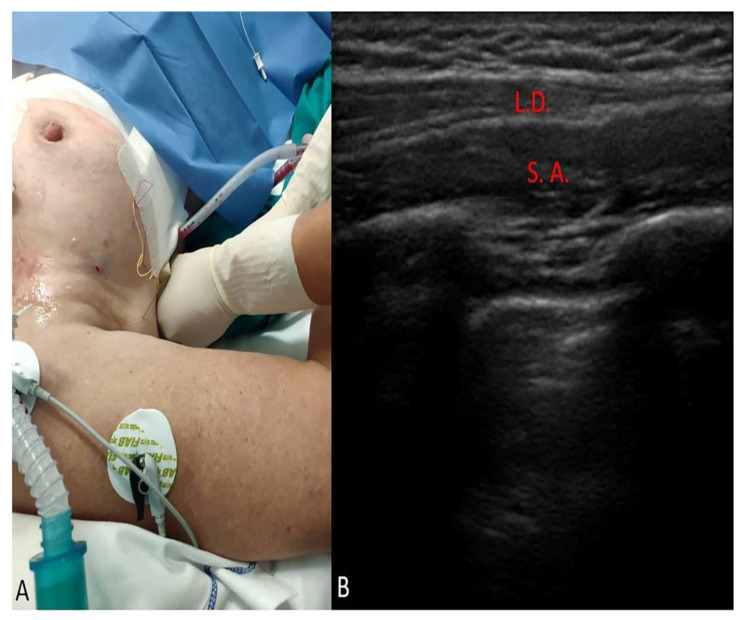
SAP block: (**A**), intraoperative positioning for the realization of the block. (**B**) ultrasound imaging scan of the inter-fascial plane deep to the anterior serratus muscle at 5th rib level in the median axillary line; L.D.: latissimus dorsi muscle; S.A.: anterior serratus muscle.

The Serratus Plane block was performed by using a Stimuplex Braun 22 G × 80 mm echogenic needle, an ultrasound linear probe (high frequency) and an ultrasound PHILIPS machine. Thirty milliliters of Ropivacaine 0.25% was injected in the inter-fascial plane deep to the anterior serratus muscle at the 5th rib level in the median axillary line (the deep approach according to Blanco et al. [14]) (Figure 3A,B). The blocks of the intercostal brachial nerves, the lateral branches of intercostal nerves (T3–T9), long thoracic nerves and thoracodorsal nerve also achieved permitting analgesia in the anterolateral region of the chest.

### 2.4. Statistical Analysis

Statistical analysis was performed using Graph Pad Prism 8.4.2 Software (GraphPad Software Inc., San Diego, CA, USA). The descriptive numerical variables (age, weight, height, BMI, starting fraction ejection) of the two groups were expressed with mean ± SD and standard error. The normality of distribution was determined for all scores using the Kolmogorov–Smirnov test. For the normally distributed data, single comparisons were performed using the Student’s *t*-test; for continuous data not normally distributed, the Mann–Whitney U test was used. Differences between two groups in the different times of the continuous variables were statistically analyzed by a two-way analysis of variance (ANOVA) mixed model followed by Tukey’s multiple comparisons test. The chi-square test or Fisher exact test was used for comparisons of categorical data classified as nominal. For all comparisons, *p* < 0.05 was considered statistically significant.

## 3. Results

The authors studied 78 patients who were extracted by the database. A total of 41 patients underwent the PECS2 block + serratus plane blocks (Group 1), and 37 underwent the intravenous opioids analgesia (Group 2). The descriptive variables of the two groups are presented in Table 1.

No difference was present between the two groups in terms of weight, height, BMI, age and starting FE. Outcome measures are summarized in Table 2.

### 3.1. Primary Outcome

**Figure 4:**
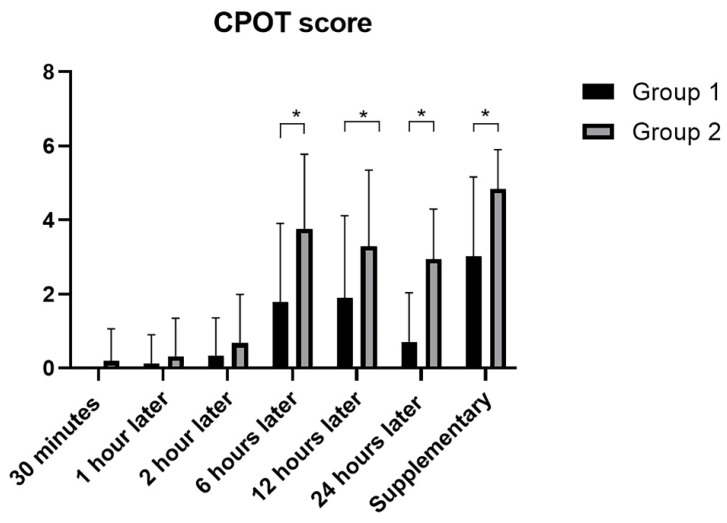
CPOT scores at different times in the two groups. *: statistically significant *p*-values.

Pain perceived at 6 h (Group 1 vs. Group 2: 1.78 ± 2.13 vs. 3.80 ± 2.10; *p* = 0.0002), 12 h (Group 1 vs. Group 2: 1.90 ± 2.21 vs. 3.30 ± 2.05; *p* = 0.0088) and 24 h (Group 1 vs. Group 2: 0.71 ± 1.33 vs. 2.95 ± 1.35; *p* < 0.0001) after start of analgesia was significantly lower in Group 1 than Group 2, and CPOT score for additional analgesia was significantly lower in Group 1 (Group 1 vs. Group 2: 3.02 ± 2.13 vs. 4.83 ± 1.1; *p* = 0.0005) (Figure 4) (Table 2). CPOT scores at 30 min (*p* = 0.2218), 1 h (*p* = 0.1844) and 2 h (*p* = 0.1959) after the start of analgesia were not statistically significant (Table 2).

**Figure 5:**
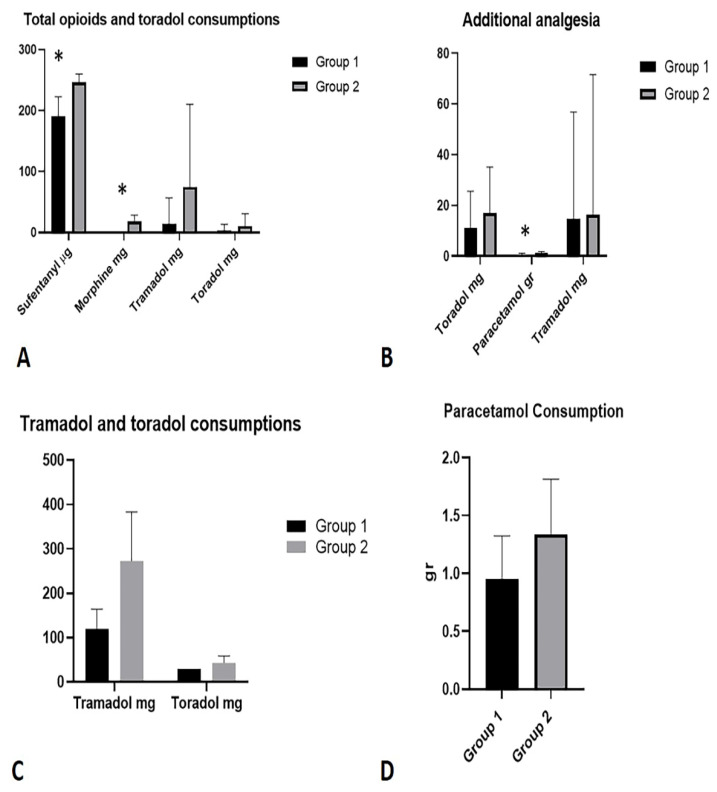
(**A**) Total opioids and toradol consumptions in the two groups. (**B**) Additional analgesia in the two groups. (**C**) Sub-analysis of tramadol and toradol consumptions in patients where they were used. (**D**) Sub-analysis of paracetamol consumption in patients where it was used. *: statistically significant *p*-values.

Comparison of data regarding total opioids consumption showed that in Group 1 was significantly lower than Group 2, except for Tramadol: Sufentanyl (µg) Group 1 vs. Group 2: 191.2 ± 31.40 µg vs. 246.2 ± 14.01 µg; (*p* < 0.0001); Morphine (mg) Group 1 vs. Group 2: 0.1220 ± 0.7809 mg vs. 18.19 ± 10.32 mg; (*p* < 0.0001); Tramadol (mg): Group 1 vs. Group 2: 14.63 ± 42.20 mg; *p* = 0.1103) (Figure 5A) (Table 2).

Furthermore, the comparison of Toradol consumption (mg) showed no statistically significant difference between Group 1 vs. Group 2: 3.659 ± 9.939 mg; (*p* = 0.2894) (Figure 5A).

The comparative analysis, with regard to additional analgesia, showed the following results in terms of the use of additional drugs (Table 2): toradol (mg): Group 1 vs. Group 2: 10.98 ± 14.63 mg vs. 17.03 ± 18.08 mg; (*p* = 0.1580); tramadol (mg): Group 1 vs. Group 2: 16.22 ± 55.34 vs. 14.63 ± 42.20 mg; (*p* = 0.9548); paracetamol (gr): Group 1 vs. Group 2: 0.5366 ± 0.5522 vs. 1.081 ± 0.6823; (*p* = 0.0004) (Figure 5B).

A sub-analysis of patients in which Tramadol and Toradol were used showed an average dosage of use, respectively: Group 1 vs. Group 2: Tramadol 120 ± 44.72 mg vs. 272.7 ± 110.4 mg; Toradol 30 ± 0 mg vs. 43.33 ± 15.81 mg (Figure 5C).

A sub-analysis of patients in whom paracetamol was used showed an average dosage of use, respectively: Group 1 vs. Group 2: 0.9565 ± 0.3666 mg vs. 1.333 ± 0.4795 mg (Figure 5D).

### 3.2. Secondary Outcome

**Figure 6:**
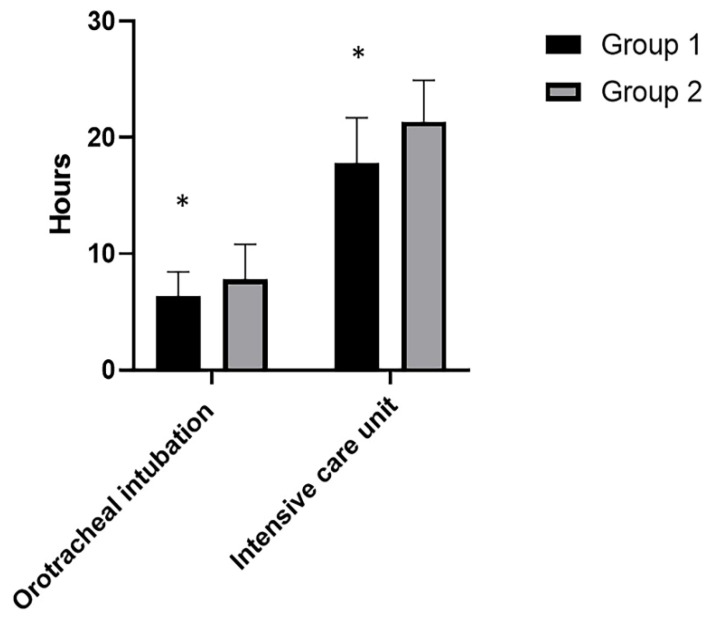
Orotracheal intubation hours in ICU and stay ICU hours in the two groups. *: statistically significant *p*-values.

Statistically significant differences in terms of hours were highlighted with regard to orotracheal intubation times in ICU (Group 1 vs. Group 2: 6.40 ± 2.08 vs. 7.81 ± 2.98; *p* = 0.0147) and also with regard to the time of stay in ICU (Group 1 vs. Group 2: 17.78 ± 3.921 vs. 21.38 ± 3.554; *p* < 0.0001) (Figure 6) (Table 2).

**Figure 7:**
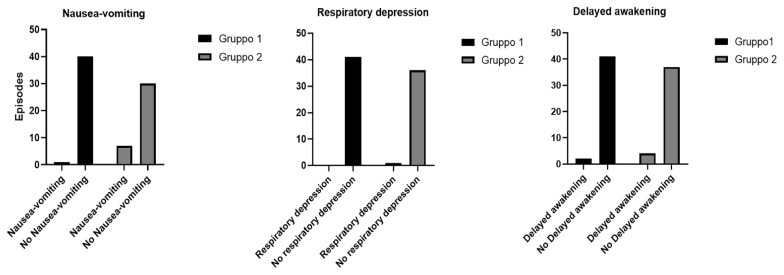
Episodes of nausea and vomiting, respiratory depression and delayed awakening in the two groups.

Comparative analyzes between the two groups on related opioid side effects showed (Table 2): episodes of nausea and vomiting (*p* = 0.0237) (Figure 7); respiratory depression (*p* = 0.2894) (Figure 7) and delayed awakening (*p* = 0.4274) (Figure 7).

## 4. Discussion

Currently, locoregional anesthesia techniques are very diffused, especially in major surgery such as colorectal and liver surgery, thoracic surgery, orthopedic surgery and kidney transplant surgery. The utility of PECS II and serratus plane block is widely demonstrated in other surgeries such as mastectomy [15], thoracic surgery [16], pacemaker implantation [17], traumatology for ribs fracture [18] and pediatric surgery [19], but there are not many studies in cardiac adult surgery. Ultrasound-guided fascial plane blocks have been embraced enthusiastically as an alternative to epidural, paravertebral and perineural injections [20]. There are several locoregional analgesia techniques in cardiac surgery pain management: peridural analgesia, paravertebral block and fascial blocks such as ESP block, serratus plane block and PECS II block.

To date, no studies have examined the PECS II block associated with serratus plane block in patients undergoing minithoracotomy in cardiac surgery.

For PECS II blocks, a study regarding analgesia in midline sternotomy [8] was performed bilaterally and compared with parental analgesia. In this study, PECS group patients required less duration of ventilator support; they had fewer pain scores and less need for rescue analgesia.

In another study about PECS II block in mini-thoracotomy in mitral/tricuspid valve repair [21], the authors associated the PECSII block with ESP block, and it was compared with a group that received only ESP block. It demonstrated that the addition of PECS blocks to ESP reduced consumption of oxycodone via patient-controlled analgesia (PCA), reduced pain intensity on the VAS and increased patient satisfaction with pain management in patients undergoing mitral/tricuspid valve repair via mini-thoracotomy.

Regarding the serratus plane block, a recent prospective observational cohort study compared the continuous SAP analgesia group with the morphine analgesia group [9]. The authors performed the deep SAP block, inserting a catheter between the serratus muscle and fifth rib because, in their opinion, the deeper approach provided simplified sonographic imaging, and there was less risk of catheter dislocation. They concluded that continuous deep serratus anterior plane block seems to be a valid alternative to intravenous opioids in terms of efficacy for patients undergoing minithoracotomy with a lower opioid requirement.

The current study set out to demonstrate that the PECS II block associated with serratus plane block is a valid alternative to intravenous analgesia and is better for some aspects. The patients who received the blocks had a reduced pain perception after 6 h, 12 h and 24 h at the start of locoregional analgesia; they consumed lower opioids quantities and used lower quantities of rescue analgesia at lower levels of pain. Furthermore, the time of stay in ICU and time of intubation was lower in patients that received PECS II and serratus plane blocks, and they had a lower number of nausea and vomiting episodes.

The association between PECS II and SAP proved itself a good choice because the two blocks acted on two different targets. PECS II block provided a better anterior chest region analgesia (the site of thoracotomic incision), while serratus plane block provided a better anterolateral chest region analgesia (the site of drainages insertion). Indeed, by serratus plane blocking the blocks of the intercostobrachial nerve (ICBN), the lateral branches of intercostal nerves (T3–T9), long thoracic nerve and thoracodorsal nerve were obtained, also permitting analgesia in the anterolateral region of the chest while the PECS II blocked the median and the lateral pectoralis nerves, long thoracic nerves, thoracodorsal nerve and lateral branches of intercostal nerves (only T2–T6).

In this study population, block-related complications were not observed (pneumothorax, nerve injury, LAST); however, these are rarely described in the literature [22].

The pain perception at 30 min, 1 h, 2 h after the start of analgesia (not statistically significant) could be determined by the long Sufentanyl half-elimination time (but that PECSII and serratus plane block used less quantity of Sufentanyl).

Finally, the time of stay in ICU was also affected by the organizational needs of the cardiac surgery unit (receiving unit) and ICU (discharging unit).

This study had several limitations. First, it was a retrospective study. However, the protocol for the association of PECS II and SAP blocks in patients undergoing minithoracotomy for aortic or/and mitral or/and tricuspid valves replacement or repair or atrial myxoma resection was standardized in the authors’ institution. Second, this study was conducted in a single institution. Finally, the study involved small samples of patients; for this, future studies including large numbers of patients will be able to contribute to better defining their utility and cost benefits.

## 5. Conclusions

In this study, compared to standard intravenous analgesia, PECS II block associated with serratus plane block was a valid alternative. The fascial blocks allowed better pain management and reduced the use of rescue analgesia, the number of episodes of collateral effects opioids related, the length of stay in ICU and the time of intubation. Furthermore, this alternative allowed to applicate the fast-track surgery improving the outcomes and comfort in patients undergoing minimally invasive cardiac surgery. Last but not least PECS II block associated with the serratus plane block appears to be an effective option to add to ERAS protocol in cardiac surgery.

## Figures and Tables

**Table 1:** Descriptive data of group 1 and group 2. FE = ejection fraction.

Group 1	Weight	Height	BMI	Age	FE at the Start	Group 2	Weight	Height	BMI	Age	FE at the Start
**Number of patients**	41	41	41	41	41	**Number of patients**	37	37	37	37	37
**Minimum**	42	152	16.8	52	42	**Minimum**	48	150	19.2	37	30
**25% Percentile**	64	160	23.15	62	53.5	**25% Percentile**	59.5	161.5	22.1	59.5	55
**Median**	74	173	25.1	71	60	**Median**	70	169	24	71	60
**75% Percentile**	83	180	26.15	77	63	**75% Percentile**	76	173	25.6	78.5	62.5
**Maximum**	119	188	51.5	85	71	**Maximum**	95	182	33.7	85	80
**Mean**	73.83	170.6	25.35	70.2	58.12	**Mean**	68.51	168.1	24.18	67.95	59.05
**Std. Deviation**	15.24	9.96	5.28	9.06	6.84	**Std. Deviation**	11.7	8.083	3.3	12.82	8.89
**Std. Error of Mean**	2.38	1.556	0.824	1.414	1.07	**Std. Error of Mean**	1.924	1.329	0.54	2.11	1.46
**Lower 95% CI of mean**	69.02	167.5	23.68	67.34	55.96	**Lower 95% CI of mean**	64.61	165.4	23.08	63.67	56.09
**Upper 95% CI of mean**	78.64	173.8	27.01	73.05	60.28	**Upper 95% CI of mean**	72.41	170.7	25.28	72.22	62.02
**Coefficient of variation**	20.64%	5.838%	20.83%	12.9%	11.78%	**Coefficient of variation**	17.08%	4.81%	13.65%	18.87%	15.06%

**Table 2:** Outcomes measures.

Variables	Group 1 (*n* = 41)	Group 2 (*n* = 37)
*CPOT score 30 min*	0 ± 0	0.18 ± 0.87
*CPOT score 1 h*	0.12 ± 0.78	0.32 ± 1.02
*CPOT score 2 h*	0.34 ± 1.01	0.67 ± 1.31
*CPOT score 6 h*	1.78 ± 2.12	3.75 ± 2.01
*CPOT score 12 h*	1.90 ± 2.21	3.29 ± 2.05
*CPOT score 24 h*	0.70 ± 1.32	2.94 ± 1.35
*CPOT score supplement analgesia*	3.02 ± 2.13	4.83 ± 1.06
*Sufentanyl (μg)*	191.2 ± 31.4	246.2 ± 14.01
*Morphine (mg)*	0.12 ± 0.78	18.19 ± 10.32
*Tramadol (mg)*	14.63 ± 42.2	81.08 ± 139.1
*Toradol (mg)*	3.65 ± 9.93	10.54 ± 20.27
*Toradol (mg) Additional analgesia*	10.98 ± 14.63	17.03 ± 18.08
*Paracetamol (mg) Additional analgesia*	0.53 ± 0.55	1.08 ± 0.68
*Tramadol (mg) Additional analgesia*	14.63 ± 42.2	16.22 ± 55.34
*Orotracheal Intubation (h)*	6.36 ± 2.08	7.81 ± 2.98
*Intensive care unit (h)*	17.78 ± 3.92	21.38 ± 3.55
*Nausea-vomiting (episodes)*	1	7
*No Nausea-vomiting (episodes)*	40	30
*Respiratory depression (episodes)*	0	1
*No respiratory-depression (episodes)*	41	36
*Delayed awakening (episodes)*	2	4
*No Delayed awakening (episodes)*	41	37

## Data Availability

The data presented in this study are available in the article.

## References

[1] Mariscalco G., Musumeci F. (2014). The minithoracotomy approach: A safe and effective alternative for heart valve surgery. Ann. Thorac. Surg..

[2] Kastengren M., Svenarud P., Ahlsson A., Dalén M. (2019). Minimally invasive mitral valve surgery is associated with a low rate of complications. J. Intern. Med..

[3] Melloul E., Hübner M., Scott M., Snowden C., Prentis J., Dejong C.H., Garden O.J., Farges O., Kokudo N., Vauthey J.N. (2016). Guidelines for Perioperative Care for Liver Surgery: Enhanced Recovery after Surgery (ERAS) Society Recommendations. World J. Surg..

[4] Wainwright T.W., Gill M., McDonald D.A., Middleton R.G., Reed M., Sahota O., Yates P., Ljungqvist O. (2020). Consensus statement for perioperative care in total hip replacement and total knee replacement surgery: Enhanced Recovery after Surgery (ERAS^®^) Society recommendations. Acta Orthop..

[5] Gustafsson U.O., Scott M.J., Hubner M., Nygren J., Demartines N., Francis N., Rockall T.A., Young-Fadok T.M., Hill A.G., Soop M. (2019). Guidelines for Perioperative Care in Elective Colorectal Surgery: Enhanced Recovery after Surgery (ERAS^®^) Society Recommendations: 2018. World J. Surg..

[6] Batchelor T.J.P., Rasburn N.J., Abdelnour-Berchtold E., Brunelli A., Cerfolio R.J., Gonzalez M., Ljungqvist O., Petersen R.H., Popescu W.M., Slinger P.D. (2019). Guidelines for enhanced recovery after lung surgery: Recommendations of the Enhanced Recovery after Surgery (ERAS^®^) Society and the European Society of Thoracic Surgeons (ESTS). Eur. J. Cardiothorac. Surg..

[7] Engelman D.T., Ben Ali W., Williams J.B., Perrault L.P., Reddy V.S., Arora R.C., Roselli E.E., Khoynezhad A., Gerdisch M., Levy J.H. (2019). Guidelines for Perioperative Care in Cardiac Surgery: Enhanced Recovery after Surgery Society Recommendations. JAMA Surg..

[8] Kumar K.N., Kalyane R.N., Singh N.G., Nagaraja P.S., Krishna M., Babu B., Varadaraju R., Sathish N., Manjunatha N. (2018). Efficacy of bilateral pectoralis nerve block for ultrafast tracking and postoperative pain management in cardiac surgery. Ann. Card. Anaesth..

[9] Toscano A., Capuano P., Costamagna A., Burzio C., Ellena M., Scala V., Pasero D., Rinaldi M., Brazzi L. (2020). The Serratus Anterior Plane Study: Continuous Deep Serratus Anterior Plane Block for Mitral Valve Surgery Performed in Right Minithoracotomy. J. Cardiothorac. Vasc. Anesth..

[10] Ritter M.J., Christensen J.M., Yalamuri S.M. (2021). Regional Anesthesia for Cardiac Surgery: A Review of Fascial Plane Blocks and Their Uses. Adv. Anesth..

[11] Devarajan J., Balasubramanian S., Nazarnia S., Lin C., Subramaniam K. (2021). Regional Analgesia for Cardiac Surgery Part 1. Current status of neuraxial and paravertebral blocks for adult cardiac surgery. Semin. Cardiothorac. Vasc. Anesth..

[12] Devarajan J., Balasubramanian S., Shariat A.N., Bhatt H.V. (2021). Regional Analgesia for Cardiac Surgery. Part 2: Peripheral Regional Analgesia for Cardiac Surgery. Semin. Cardiothorac. Vasc. Anesth..

[13] Constantin V., Carap A.C., Zaharia L., Bobic S., Ciudin A., Brătilă E., Vlădăreanu V., Socea B. (2015). High correlation of lung ultrasound and chest X-ray after tube drainage in patients with primary spontaneous pneumothorax: Can we omit X-rays for tube management?. Eur. Surg..

[14] Blanco R., Parras T., McDonnell J.G., Prats-Galino A. (2013). Serratus plane block: A novel ultrasound-guided thoracic wall nerve block. Anaesthesia.

[15] Al Ja’bari A., Robertson M., El-Boghdadly K., Albrecht E. (2019). A randomised controlled trial of the pectoral nerves-2 (PECS-2) block for radical mastectomy. Anaesthesia.

[16] Chong M., Berbenetz N., Kumar K., Lin C. (2019). The serratus plane block for postoperative analgesia in breast and thoracic surgery: A systematic review and meta-analysis. Reg. Anesth. Pain Med..

[17] Yang J.K., Char D.S., Motonaga K.S., Navaratnam M., Dubin A.M., Trela A., Hanisch D.G., McFadyen G., Chubb H., Goodyer W.R. (2020). Pectoral nerve blocks decrease postoperative pain and opioid use after pacemaker or implantable cardioverter-defibrillator placement in children. Heart Rhythm..

[18] Martinez T., Belveyre T., Lopez A., Dunyach C., Bouzit Z., Dubreuil G., Zetlaoui P., Duranteau J. (2020). Serratus Plane Block Is Effective for Pain Control in Patients with Blunt Chest Trauma: A Case Series. Pain Pract..

[19] Kaushal B., Chauhan S., Saini K., Bhoi D., Bisoi A.K., Sangdup T., Khan M.A. (2019). Comparison of the Efficacy of Ultrasound-Guided Serratus Anterior Plane Block, Pectoral Nerves II Block, and Intercostal Nerve Block for the Management of Postoperative Thoracotomy Pain after Pediatric Cardiac Surgery. J. Cardiothorac. Vasc. Anesth..

[20] Black N.D., Stecco C., Chan V.W.S. (2021). Fascial Plane Blocks: More Questions Than Answers?. Anesth. Analg..

[21] Gawęda B., Borys M., Belina B., Bąk J., Czuczwar M., Wołoszczuk-Gębicka B., Kolowca M., Widenka K. (2020). Postoperative pain treatment with erector spinae plane block and pectoralis nerve blocks in patients undergoing mitral/tricuspid valve repair—A randomized controlled trial. BMC Anesthesiol..

[22] Balan C., Bubenek-Turconi S.I., Tomescu D.R., Valeanu L. (2021). Ultrasound-Guided Regional Anesthesia-Current Strategies for Enhanced Recovery after Cardiac Surgery. Medicina.

